# Spinal primitives and intra-spinal micro-stimulation (ISMS) based prostheses: a neurobiological perspective on the “known unknowns” in ISMS and future prospects

**DOI:** 10.3389/fnins.2015.00072

**Published:** 2015-03-20

**Authors:** Simon F. Giszter

**Affiliations:** ^1^Department of Neurobiology and Anatomy, Drexel University College of Medicine, Drexel UniversityPhiladelphia, PA, USA; ^2^School of Biomedical Engineering and Health Systems, Drexel UniversityPhiladelphia, PA, USA

**Keywords:** intraspinal microstimulation (ISMS), vector superposition, primitives, pattern generators, spinal motor maps

## Abstract

The current literature on Intra-Spinal Micro-Stimulation (ISMS) for motor prostheses is reviewed in light of neurobiological data on spinal organization, and a neurobiological perspective on output motor modularity, ISMS maps, stimulation combination effects, and stability. By comparing published data in these areas, the review identifies several gaps in current knowledge that are crucial to the development of effective intraspinal neuroprostheses. Gaps can be categorized into a lack of systematic and reproducible details of: (a) Topography and threshold for ISMS across the segmental motor system, the topography of autonomic recruitment by ISMS, and the coupling relations between these two types of outputs in practice. (b) Compositional rules for ISMS motor responses tested across the full range of the target spinal topographies. (c) Rules for ISMS effects' dependence on spinal cord state and neural dynamics during naturally elicited or ISMS triggered behaviors. (d) Plasticity of the compositional rules for ISMS motor responses, and understanding plasticity of ISMS topography in different spinal cord lesion states, disease states, and following rehabilitation. All these knowledge gaps to a greater or lesser extent require novel electrode technology in order to allow high density chronic recording and stimulation. The current lack of this technology may explain why these prominent gaps in the ISMS literature currently exist. It is also argued that given the “known unknowns” in the current ISMS literature, it may be prudent to adopt and develop control schemes that can manage the current results with simple superposition and winner-take-all interactions, but can also incorporate the possible plastic and stochastic dynamic interactions that may emerge in fuller analyses over longer terms, and which have already been noted in some simpler model systems.

## Introduction

The goal of this review is to bring together our current neurobiological understanding of spinal organization, and of motor modularity, together with the needs and issues arising in designing and implementing an intraspinal microstimulation (ISMS) neuroprosthetic device. We then seek to identify several areas in which there is a clear lack of knowledge, and more specifically the needs in these areas that are essential for truly effective spinal prosthetic devices to be developed. The emphasis is on segmental motor controls. However, the ramifications of the analysis likely apply also for autonomic controls and other applications, as will be touched on.

ISMS as a therapeutic strategy holds great promise. The target motor pools and interneuronal control systems for most aspects of segmental motor controls are all present in spinal cord, and they are arranged in various topographic structures. Sympathetic and parasympathetic systems are also arranged topographically in the cord and must integrate with the motor organization for optimal function in able-bodied individuals. Epidural stimulators and spinal nerve stimulation already have extensive applications, and in part they exploit these topographic features. Novel designs and applications of such epidural stimulation are being developed in basic research contexts (Courtine et al., 2009; Gerasimenko et al., 2010; Hsieh and Giszter, 2011; Wenger et al., 2014) and in the clinic (Harkema et al., 2011; Angeli et al., 2014; Sayenko et al., 2014). Magnetic stimulation also shows interesting opportunities (Sasada et al., 2014). Intraspinal stimulation might further improve on the precision of epidural and magnetic methods, though at some risk, especially because of the need to implant and thereby breach the dura and blood brain barrier. However, instraspinal stimulation offers the greatest possibility of selectivity and precision of control of pattern generation, or motor pools (Saltiel et al., 1998; Mushahwar and Horch, 2000; Mushahwar et al., 2004; Barthelemy et al., 2006, 2007; Holinski et al., 2011; Sunshine et al., 2013; Mondello et al., 2014) and perhaps it may allow new modes of prosthetic control, by using primitives in the modular spinal motor hierarchies in more subtle ways (Bizzi et al., 1991, 2008; Giszter et al., 1993; Mussa-Ivaldi et al., 1994; Tresch and Bizzi, 1999; Lemay et al., 2001; Lemay and Grill, 2004). However, there remain very significant hurdles to realizing this promise, in the theoretical, the basic research and the technical engineering areas of ISMS applications.

As knowledge of spinal motor structures and their development has increased, a range of functional targets and potentially exploitable control structures have gradually been revealed. Spinal circuitry has significant capacity to organize simple behaviors, in part independently of the brain. These circuits may lighten the computational load of descending systems. These circuits include the pattern generation systems (“central pattern generators,” abbrev. CPGs, see below for more detail) for repetitive behaviors such as locomotion, which are hierarchically organized into rhythm generation and pattern shaping circuitry, and also sets of modular building blocks or primitives acting as compositional elements in routine motor acts. These circuits are all found experimentally across all legged (tetrapod) vertebrate species examined, and these are also sometimes accessible to microstimulation. Targeting these systems in the spinal cord (via epidural or ISMS approaches) offers the possibility of exploiting this intrinsic control organization, more or less as the brain does. It may then be feasible to think of recruiting motor pools either in patterned sequences, in natural synergies, or as fractionated pools, with more normal recruitment orders and reflex engagement than found in classical peripheral functional electrical stimulation (FES) approaches. These various features and advantages suggest that ISMS should be the basis of effective clinical therapies, but it is clearly not at this time. Several things have thus far impeded the effective translation to clinic as discussed in detail below. First, and maybe the major issue, electrodes or optrodes that can survive and operate robustly in the spinal environment for ISMS and recording are not readily available. This is because the spinal cord is likely in many ways the most hostile CNS environment for chronic recording and stimulation, due to the motion ranges experienced there. However, a second set of issues that currently limit translation are related to integrating our knowledge of the spinal neurobiology's motor hierarchies and their operations together with the current electrodes' capabilities. Ideally, we need to achieve a robust control in ISMS that is based both on the neurobiology and also on an understanding of the interactions of ISMS with other therapies and the injury type, e.g., spinal cord injury (SCI) and rehabilitation interactions. In this review we focus on these areas and the limitations in our current knowledge.

## Neurobiological understanding of spinal controls, and possible affordances for ISMS

What targets and mechanisms which are intrinsic to spinal cord are available to ISMS for organizing motor action? How do these operate normally in the intact system? Work over the last 25 years, that began with ISMS tests in frogs, supports a modular compositional mechanism for discrete motor actions that is resident in the spinal cord (Bizzi et al., 1991; Giszter et al., 1993, 2007; Loeb et al., 1993; Mussa-Ivaldi et al., 1994; Saltiel et al., 1998; Kargo and Giszter, 2000; Hart and Giszter, 2004, 2010; Cheung et al., 2005; Kargo et al., 2010; Giszter and Hart, 2013). The framework suggested by this body of research is one whereby a small number of motor primitives support a core of spinally organized reflex and rhythmic behaviors, these primitives operating under the control of the CPGs' rhythm generation and pattern shaping systems. This modular organization and the evidence for it is worth reviewing and comparing across species here, because the organization and rules under which these circuits operate may either enhance or interfere with ISMS strategies in neuroprosthetics. The core spinal organization that supports modules or primitives appears conserved across limbed vertebrate species. Data in support of modularity has been gathered in many preparations. The analyses have been designed using various methods, and data span from frogs (ibid.), and neonatal mice preparations during development (Levine et al., 2014) to intact cats (Krouchev et al., 2006; Drew et al., 2008; Krouchev and Drew, 2013) and humans (Ivanenko et al., 2007, 2013; Chvatal et al., 2011; Dominici et al., 2011), and include clinical observations (Cheung et al., 2009, 2012; Clark et al., 2010; Chvatal et al., 2013; Hayes et al., 2014). Despite this wealth of data there remain lots of “known unknowns” regarding these systems. We first review the relatively established “knowns”.

**CENTRAL PATTERN GENERATORS (CPGs):**

Operationally defined circuits that can generate patterned motor activity closely resembling a normal pattern, in the absence of patterned input or sensory feedback. Classically, neurobiologists identify CPGs by paralysis or deafferentation of a animal model system.

The CPG circuits for limb control are now often divided conceptually into:-

**(1) RHYTHM GENERATION CIRCUITS-** driving the overall rhythm frequency and cadence.

**(2) PATTERN FORMATION CIRCUITS-** circuitry organizing the detailed bursts and precise muscle composition in the executed pattern and paced or driven by the rhythm generator.

### Early ISMS data was a signpost to interesting neurobiological data

**Figure 1:**
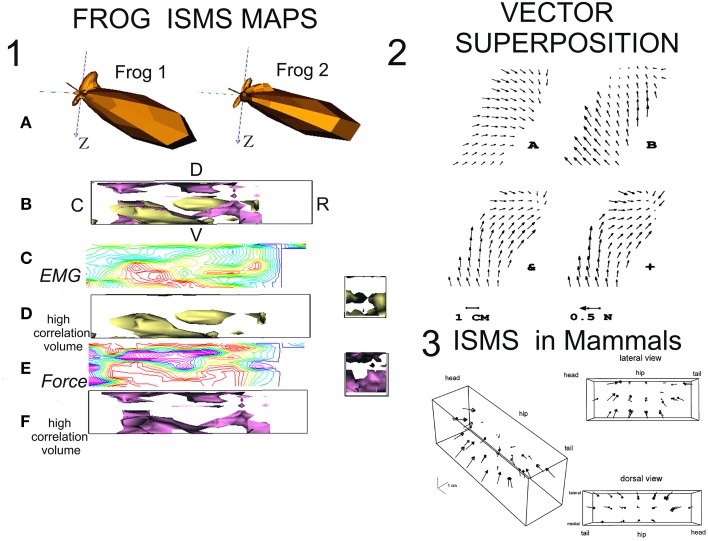
**Mapping of ISMS results and superposition in frogs and rats**. **(1)** ISMS maps in lumbar spinal cord of spinal frogs. Redrawn from Giszter et al. (2000) with permission. (A) Polar spherical plots of the force directions recruited by ISMS. There is high similarity of 3D isometric ankle force recruitments across the spinal cords of two test maps in spinalized frogs. (B) 3D Map of lumbar cord with orientation and overlay of data in D and F (R-rostral; C-caudal; D-dorsal; V-ventral). The surfaces shown enclose regions of higher ISMS correlation between frogs. (C) A sample contour heat map of ISMS EMG (vector) recruitment correlation across the map in a sagittal plane at 400 microns from the midline. Red high, blue/magenta low. (D) Surface enclosing volumes of higher correlation interfrog regions in the ISMS EMG (vector) responses. (E) A sample contour heat map of ISMS force recruitment direction correlations across the map in a sagittal plane at 400 microns from the midline. Red high, blue/magenta low. (F) Surface enclosing volumes of higher correlation interfrog regions in the ISMS force direction responses. **(2)** Vector superposition of ISMS results (Mussa-Ivaldi et al., 1994) with permission. Two differing fields recruited by ISMS can be combined by co-stimulation to generate vector superposition. (A) Site 1 alone. (B) Site 2 alone. &, the result of co-stimulation; +, the vector sum of A + B. The fields in + and & are almost identical. **(3)** The force field primitives/motor primitives/EMG synergies seen in frog can be recruited in mammals (cat, rat). Here a complete 3D isometric force field obtained from lumbar ISMS in Tresch and Bizzi (1999) is shown with permission.

ISMS in spinalized frog, applied throughout intermediate zone of the spinal cord recruits a few modules (Bizzi et al., 1991; Giszter et al., 1993; Mussa-Ivaldi et al., 1994). These modules comprise groups of muscles acting as synergies, and the monosynaptic feedback pathways, and together these all act as well integrated units with clearly defined biomechanical effects. There seem to be a limited number of modules in the spinal cord. There is also a rough topography in some species (see Figure 1, Panel 1 for examples of modular force directions and topography data from ISMS in two spinal frogs). The modules comprise muscles spanning multiple joints activated synchronously in fixed ratios and the net effect of these modules is to generate whole limb force/torque patterns which vary systematically over the work space and, under isometric conditions, these can be thought of as isometric force-fields at the ankle or limb endpoint and directly measured. This force-field framework essentially represents the effects of limb and muscle biomechanics, coupled directly with the action of a set of selective and modular interneuronal drives to motor pools. This modularity has now also been examined (see below) in various species via neural recordings (Hart and Giszter, 2010), and molecular genetics (Levine et al., 2014). The selective drives generating the modularity could be recruited in quiescent frog spinal cord with ISMS. More interesting was the result of co-stimulation of sites recruiting different modules (see Figure 1, Panel 2 for examples of co-stimulation results). In 80–85% of tested sites it was discovered that ISMS of multiple sites in the frog spinal cord generated a linear superposition of the force-fields recruited separately at each site (Mussa-Ivaldi et al., 1994). The ISMS recruited synergies and force-field patterns could then be thought of as a basis set for the spinal cord, or for a device controlling the limb through ISMS (see e.g., Mussa-Ivaldi and Giszter, 1992; Lemay et al., 2001). The co-stimulation experiments indicated that the ISMS superposition and modularity results together could be used as a compositional system to artificially construct force patterns in the limb, using modules drawn from the basis set (otherwise termed a collection of primitives) embedded in spinal cord. This was definitively demonstrated as an ISMS control method in frog by Lemay et al. (2001). ISMS in rats also showed similar modular organization (Tresch and Bizzi, 1999), e.g., see Figure 1, Panel 3. At the same time Mushahwar and colleagues explored recruitment of individual motor pools using ISMS among motoneurons in the deeper ventral horn in the cat (Mushahwar and Horch, 1998, 2000), and showed more normal recruitment orders than from peripheral FES for single muscle recruitments. The future of ISMS at the time thus looked very promising, and at the same time ISMS data led to new kinds of neurobiological analyses and directions. ISMS suggested new ways to think about spinal movement construction.

**MOTOR PRIMITIVES:** unitary assemblies of muscles (or 'muscle synergies') in space or time or both. In this article we focus on pulsed spatial synergies.

**SPATIAL SYNERGIES:** strongly covarying muscles recruited in fixed ratios to one another.

**FORCE-FIELD PRIMITIVES:** the biomechanical force effects of a spatial synergy muscle group activation measured or calculated across the limb configuration space. ISMS may recruit Force-Field primitives.

**TEMPORAL SYNERGIES:** strongly covarying pulses (or bursts) of muscle activation, within a motor pattern, which may be of constant duration in some cases. The muscle composition may vary in a temporal synergy.

### Neurobiological explorations of modularity adopting the primitives seen in ISMS

**Figure 2:**
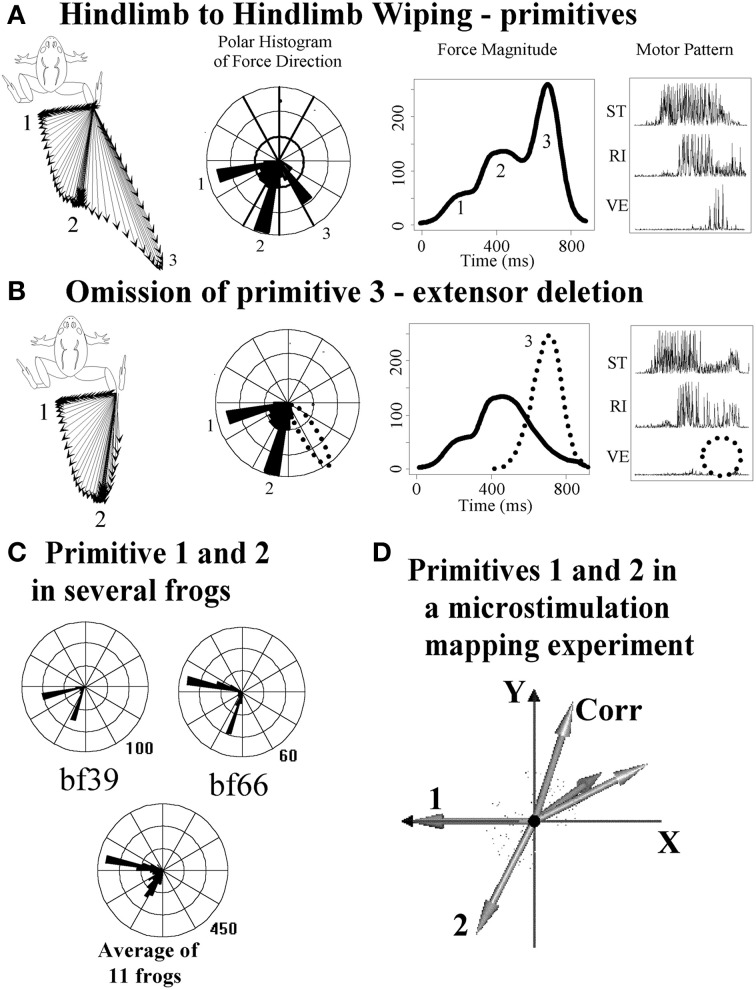
**Correspondence of ISMS force direction clusters to primitives recruited and deleted from natural behaviors**. Data re-plotted from Giszter and Kargo (2000). **(A)** Hindlimb wiping with the limb held isometrically results in three generation of force directions (vector plot and polar histogram) and three overlapping force pulses (magnitude plot) with associated EMG. **(B)** Omission (i.e., deletion as per Stein) of phase 3 and phase 2 can occur. Here phase 3 is lost. The effect is to remove a force pulse in a specific direction and an EMG synergy from the wiping pattern. **(C)** These effects are consistent across frogs, as seen in polar histograms. **(D)** The polar histograms in **(C)** correspond to directions elicited by ISMS (e.g., in the maps in Figure 1, panel 1). Primitive 1 and 2 in **(D)**, from ISMS, correspond to the polar histogram directions in several frogs in **(C)**, suggesting that natural primitives/modules are recruited by ISMS. Corresponding with the deletions of synergies/primitives shown here, there are also added-in corrections (Kargo and Giszter, 2000) showing superposition of synergies/primitives is a general method of spinal motor pattern construction.

**Figure 3:**
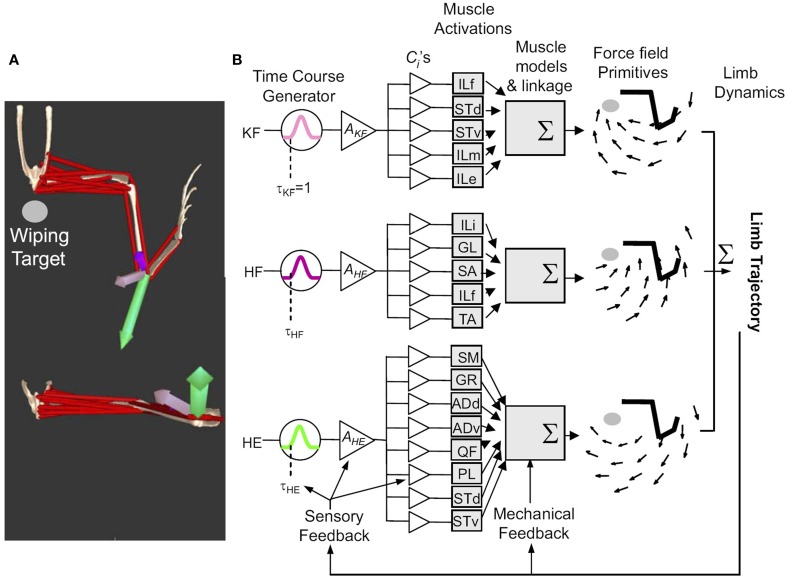
**The general framework of movement construction suggested from** Figure 2. Figure reproduced from Kargo et al. (2010). **(A)** Reflex behaviors are organized as collections of primitives which can be characterized as having different force patterns and differing force vectors at a given location. Opensim Model data can reproduce this from a model based on synergies and premotor drives. **(B)** Primitives (for wiping 3, KF, HE, HF) comprise pulsed activation of drives (e.g., time course τ_KF_, peak activation A*_KF_*) which activate muscles in balances given by the fixed c*_i_*, determined by the interneuron drive projections. Individual muscle forces sum to determine a visoelastic force-field (i.e., torque/force pattern driving the limb and environment interaction). Limb dynamics are driven by this and feedback systems work so as not to violate this modular structure.

**Figure 4:**
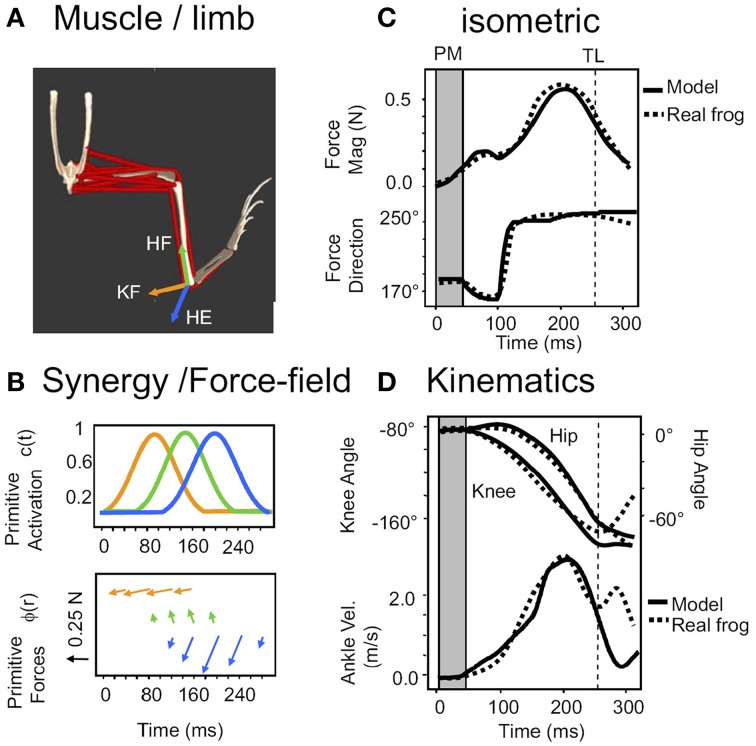
**The system of reflex and ISMS activated primitives is sufficient to accurately model reflex generated behaviors**. Reproduced from Kargo et al. (2010) with permission. **(A)** The simulation scheme in Figure 3 is implemented in Opensim as a frog biomechanical model. **(B)** Combinations of pulsed synergies driving force-fields, sequenced by a combination of central and afferent feedback modulations of pattern organize limb force/torque patterns. **(C)** These can replicate the force production under isometric conditions (dotted lines-measured data of a spinal bullfrog, solid lines- opensim prediction). **(D)** Given the viscoelastic model of muscle and afferent feedback based reduction of pulse activations in a moving system the open sim model reproduces joint kinematics and ankle (effectively the tool) motion in the wipe task. This suggests ISMS can be used to organize motion according to the same rules as natural reflex actions, modulo the concerns noted in Section *A Program of ISMS Research Based on the Current “Known Unknowns*.”

The ISMS generated effects in Bizzi, Mussa-Ivaldi, and Giszter's experiments could in principle have been driving the spinal cord in ways far from the normal spinal operation. However, clues that ISMS results were not spurious, but rather physiologically relevant, were found in the 15–20% of ISMS trials where linear superposition was violated. Violations were all winner-take-all. Further, we found that the force patterns recruited by ISMS resembled those in natural behaviors elicited from spinal cord, e.g., wipe or scratch behaviors (Giszter et al., 1993). Paul Stein had also shown merging, blending, winner-take-all and modular deletion rules operated in central pattern generation in the turtle swimming and scratching systems (Stein et al., 1986; Stein, 2008). ISMS results and combination rules matched with the merging, blending and deletion rules of operation that Stein first revealed in turtle. Further careful examination of frog reflex behaviors subsequently also showed that trajectory construction and corrections controlled by the spinal cords of frogs matched exactly with the primitive vector superposition and combination mechanisms identified from ISMS (Kargo and Giszter, 2000). In addition, Stein-type deletions occurred in frogs (Giszter and Kargo, 2000). See Figure 2 for examples. In the natural behaviors organized in spinal cords of frogs it was also observed that each primitive had a characteristic timescale i.e., a fixed pulsed structure (Kargo and Giszter, 2000; Hart and Giszter, 2004). In effect, the careful myography and measurements showed that the spinal cord composed the precisely targeted trajectories in its reflex behaviors by using pulsed combinations of primitives, and the spinal cord then corrected these trajectories by similar means if they were perturbed. This further simplified the motion construction strategy in spinal systems. Attempts to alter the pulses or primitives failed (i.e., break the temporal and spatial synergy constraints). Using proprioceptive vibration of individual muscles it was possible to amplify and change the phase of pulses, but not to break them up (Kargo and Giszter, 2008). Further, the same amplification and phase mechanisms, that were identified as proprioceptive effects, could in simulations be combined with proprioceptive recordings patterns and limb mechanics to provide a compact neurobiological and biomechanical model which accounted quite accurately for the wipe trajectory formation and variation across the limb workspace (Kargo et al., 2010), as shown in Figures 3, 4. In effect, neurobiological analyses showed a collection of spinal primitives to construct reflex behaviors could be identified in the frog. These were based on systematic recruitment of a few (spatial) muscle synergies in well-defined pulsed patterns. It was natural next to ask if there was a defined interneuronal apparatus dedicated for this strategy, or were modules an emergent structure of more general connections? Neural recordings showed specific subsets of frog interneurons with mono or disynaptic connections to motor pools that had projection patterns which matched the modules discovered from reflex behavior manipulations (Hart and Giszter, 2010), and from blind source separation analysis of electromyogram (EMG) signals in spinal and less reduced frogs (Hart and Giszter, 2004; d'Avella and Bizzi, 2005; Roh et al., 2011).

Modular patterning and deletions similar to those in frogs and turtles were also identified in data from fictive locomotion in cat neurophysiology and these mammalian data were used to establish a separation of rhythm generation and pattern shaping in the locomotor CPGs operating in the spinal cords of the cat (Rybak et al., 2006; McCrea and Rybak, 2007, 2008). The use of various modular decomposition methods subsequently suggest that very similar motor compositionality existed across species, and also in disease processes and trauma, e.g., in cats (Krouchev et al., 2006; Drew et al., 2008; Krouchev and Drew, 2013), in humans (Ivanenko et al., 2005, 2007, 2013; d'Avella et al., 2006, 2008; Torres-Oviedo et al., 2006; Torres-Oviedo and Ting, 2007, 2010; Chvatal et al., 2011; Dominici et al., 2011; Berger et al., 2013) and in clinical observations (Cheung et al., 2009, 2012; Clark et al., 2010; Chvatal et al., 2013; Fox et al., 2013; Hayes et al., 2014). Taken together, these data and ISMS data supported the idea that ISMS might provide a means to control the spinal cord motor apparatus simply and in a biomimetic fashion to restore some functions and exploit spinal circuitry more fully.

### Controlling motion in a modular fashion using primitives—neurobiology and theory

Partly inspired by the ISMS data obtained in frogs, Mussa-Ivaldi also developed a theoretical framework based around combinations of a conservative force field basis and a circulating force-field basis (Mussa-Ivaldi, 1992; Mussa-Ivaldi and Giszter, 1992). The basic idea is that any arbitrary smooth force patterns can be straight forwardly approximated with appropriate combinations of the two bases. However, in practice, in the experiments in living vertebrate systems, it was found that ISMS and reflex activation never activated any strongly circulating force patterns. Circulation effects were usually at the measurement noise levels. The biological basis set of primitives in spinal cord are thus restricted to the conservative field basis. This restriction limits the types of fields that may be approximated. However, this restriction in biology to the conservative field basis set also brings the benefit that the biological patterns are necessarily stable in interaction with arbitrary passive environments (see work of Colgate and Hogan, 1988). In summary, instead of setting up a mechanics that intrinsically has a circulation, and is energy generating, but that risks unstable interaction, [as actually happens in many invertebrates (e.g., see Josephson et al., 2000)], the limbs of vertebrates appeared to cycle through sequences of conservative force-field (ergo locally stable) states. This cycling through sequences of stable primitives is presumably the job of the spinal CPGs and their rhythm generation and pattern shaping circuitry. These CPG circuits would recruit the primitives needed, which are then combined through the superposition mechanisms. In practice, the muscle properties in dynamic conditions impart viscous as well as elastic properties. Accordingly, in a force-field description of the motor output, in practice the force-fields generated are actually conservative viscoeleastic structures. The biological set of viscoelastic primitives can also be considered to form members of the classes of contracting systems (Slotine and Lohmiller, 2001; Richardson et al., 2005a,b). Contracting systems represent a broader scoped stability constraint, for example extending to oscillations.

Following the initial Mussa-Ivaldi formulation, the modular force production ***F*** in the frog spinal system can be written as conservative viscoelastic fields:

(1) $$F(q,\;\dot{q})\;=\;\sum_{i}A_{i}\Phi_{i}(q,\;\dot{q})\;+\;\sum_{i}B_{i}\Theta_{i}(q,\;\dot{q})$$

Where the **Φ***_i_* are the conservative basis fields and the Θ*_i_* are circulating fields which are effectively all zero.

For motion synthesis and adaptation in this modular framework, the problem can then be described as that of estimating an appropriate forcing and impedance approximation. We can write limb or body dynamics:

(2) $$M(q)\ddot{q}\;+\;G(q,\;\dot{q})\;+\;E(q,\;\dot{q},\;t)\;=\;F(q,\;\dot{q},\;t)$$

Where ***M(q)*** represents inertial terms, ***G*** interaction terms, ***E*** environmental forces, and ***F*** the torque generation needed for the task or adaptation by the musculo skeletal plant.

Following the approach of Mussa-Ivaldi (1992), and Equation (1), the activity of the modular recruitment of muscles and feedback pathways working as modular groups or primitives in pulsed activations (as clearly are shown to do in frogs) can then be generically represented as a superposition of multidimensional time-varying conservative force-field pulses in joint space. The problem can be rewritten and represented:

(3) $$F(q,\;\dot{q},\;t)\;=\;C(q,\;\dot{q},u(t))\;=\;\sum_{i}A_{i}a_{i}(t\;+\;\tau_{i})\Phi_{i}(q,\;\dot{q})$$

Where *q_i_*, $\dot{q}$, are joint angles and angular velocities, and *t* is time, *F* is the field expressed as joint torques, in general joint coordinates, *u(t)* is the applied control in muscle activations and *C* is a (non-invertible) function transforming muscle activations to forces *F*. Spinal motor primitives then provide a modular basis for constructing the potentially arbitrary force patterns required. Each primitive is a visoeleastic field Φ*_i_* activated with amplitude *A_i_* as a pulse of shape and duration given by function *a_i_*(*t*), at phase ***τ**_i_*. [Rightmost equality in Equation (2)]. Effectively the control *u*(*t*) needed is reduced (as on the right) to an appropriate set of selections of *A_i_ and τ_i_* for basis fields Φ*_i_*needed to achieve the requisite approximation, considering *a_i_*(*t*) fixed.

This framework is demonstrably effective for frog wiping behaviors, and corrections of these, where all *a_i_*(*t*) are similar to one another (Kargo and Giszter, 2000; Kargo et al., 2010), as shown in the examples of Figures 3, 4, and for quiescent frog ISMS (Mussa-Ivaldi et al., 1994).

**Figure 5:**
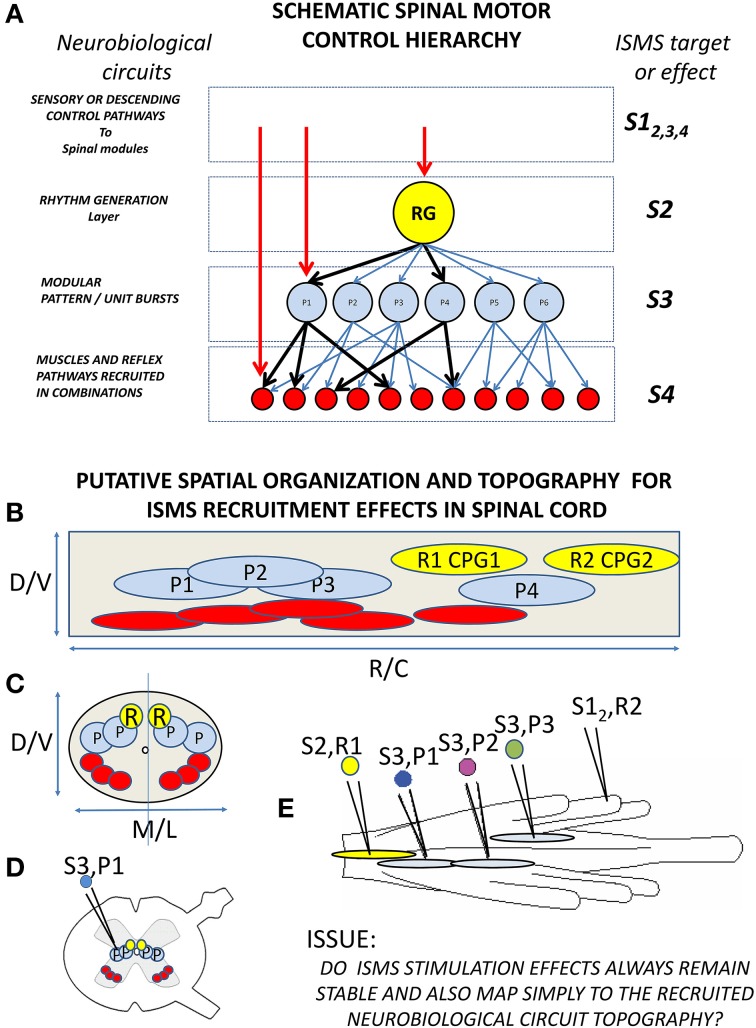
**Schematic of neurobiological hierarchies and modularity in spinal cord, and topographic organization and ISMS. (A)** *The current understanding of the spinal motor hierarchy in modular terms*. On the left are the neurobiological component labels. A rhythm generator and pattern shaper layer form the CPG. In, or just below the pattern shaping layer are the modular motor primitives which recruit specific motor pools in specific balances (e.g., heavy black lines). On the right are levels of ISMS stimulation targeting into this hierarchy: S1 stimulation of afferent or descending controls into the hierarchy (e.g., reticulospinal drive, or afferent feedback to synergists); S2 stimulation of rhythm generation; S3 stimulation of motor primitives; S4 stimulation of motor pools. **(B,C)** *Schematic topography of circuitry and best targets of ISMS in spinal cord (These need not be identical)*. **(B)** Lateral or sagittal view of cord gray matter (cell bodies and dendrites). **(C)** Cross section of cord gray. CPG rhythm centers (yellow), synergies/primitives (blue) and motor pools (red) are organized both along the cord and at different depths and mediolateral locations. **(D)** Gray matter and targets in white matter in a more realistic cross section. **(E)** *Top view of a frog cord with different targets and the relations of importance for ISMS interactions*. S2,R1: stimulation of CPG rhythm generation R1 in gray matter. S3,P1 and S3, P2: stimulation of primitives in gray matter on same side of cord (expected vector superposition). We do not know if S3,P1 and S3,P2 stimulation operate similarly when S2, R1 is activated in parallel. S3,P3: stimulation of a contralateral primitive in spinal cord. This might alter superposition of S3,P1 and S3,P2 and may not show vector superposition with S3,P1 alone. S1,R2 stimulation of afferent driving a CPG rhythm generator R2. We do not know if ISMS (e.g., S2,R1) or afferents (S1,R2) take precedence, or how S1,R2 affects the other stimulation responses (e.g., S3,P1). Minimally, we need an understanding of these interactions and topographic effects to drive an isolated spinal cord effectively with ISMS. Each may also have autonomic sequelae.

Is this analysis and the neurobiological data discussed a sufficient basis for design of an effective working motor prosthesis in, e.g., SCI? Conceptually, the work reviewed to date suggests a scheme as summarized in Figure 5. Figure 5A summarizes the neurobiological concepts underpinning the scheme, including pattern generation and primitives. In addition, on the right of the panel it suggests the points in this scheme that ISMS or optrodes might “tap into” these circuits. The observations of neurobiological topography and of ISMS topography in the quiescent or resting spinal cord are summarized in 5B in a sagittal axis view of the spinal cord and in 5C in cross section. CPG and primitive and motor pool circuits and cell body recruitment by ISMS may form a topography that gives focused control. For example in 5D stimulation in intermediate zone interneuron systems may recruit primitives. In 5E several different kinds of stimulation are indicated- stimulation of CPG level circuits, of ipsilateral primitives, of contralateral primitives, and recruitment of CPG systems through afferent stimulation. These various interactions must be well understood and defined in a fail-safe device.

#### Are the spinal primitives and circuitry a sufficient basis for a neuroprosthesis?

It is reasonable to ask first if the spinal basis of primitives as defined above will actually be sufficient for activities of interest. Current ideas suggest that the core primitives that are found may in practice have evolved so as to be optimal ways to excite limb mechanics, albeit coarsely, given the limb structure and the repertoire of the animal (Berniker et al., 2009). The modules allow coarse direct development of an initial behavior set from the primitives, but may be insufficient for refined or more novel and inventive motions. Likely both the core primitive basis and supplementing muscle activity around this are used in skilled intact volitional behaviors, even in frogs. It is now known that some ISMS and epidural stimulation can also directly activate rhythm generation systems, e.g., in the cat (Barthelemy et al., 2006, 2007), thus providing access at each level shown in Figure 5. The access to pattern systems coupled with the ISMS based recruitment of primitives might expand the flexibility and adaptation of control by engaging additional circuits and spinal computations. In summary, basic gait and some adjustments might be driven using core primitives and/or rhythm generator in an ISMS neuroprosthetic device recruiting primitives, but a “neuroprosthetic foxtrot” would likely require both the core primitive controls, the rhythm generator control and likely additional fractionated muscle recruitments besides.

#### Topography–should we expect to systematically recruit primitives with ISMS?

Motor pool topography is well understood and develops very systematically (Tsuchida et al., 1994), and afferents and interneuron systems are also developed in systematic topographies related to these (Pfaff et al., 1996; Levine et al., 2012). The interneurons associated with primitives might be expected to be topographically organized and accessible, and in neonates are (Levine et al., 2014). In the frog it was possible to create repeatable maps of spinal responses to ISMS and map locations to recruit primitives (Giszter et al., 2000) albeit coarsely. Thus, maps of motor pools, interneurons and projections should at least in principle allow systematic access to fractionated and synergy based motor controls.

### Neurobiology moves on

From the neurobiological point of view the usefulness of ISMS was primarily as a tool for focused spinal activation, and of limited scope. Issues of the normal (and broad) neural dynamics vs. a locally focused ISMS activation, propagation of unusual activation patterns, and for the focused ISMS activation the lack of specificity in locally activating excitatory or inhibitory cells and fibers of passage all limit the ways in which ISMS alone can convincingly tests hypotheses about natural modularity, spinal organization and circuit function. Many significant neurobiological questions were not directly accessible using ISMS. Better neurobiological tools exist to attack many of the interesting questions in modular motor control, and thus these were instead addressed by other means (e.g., Kargo and Giszter, 2000; Hart and Giszter, 2010; Kargo et al., 2010). However, there are serious sets of concerns about ISMS based prostheses that are often also directly related to these neurobiological limitations of ISMS, as well as limits of the newer ISMS results. Optrode methods using optogenetics can finesse only some of these issues, and will also often face the same concerns discussed below regarding compositionality, superposition and plasticity. We do not know if particular topographies observed early in development actually help in improving access to the spinal circuits via ISMS in adult animals. We often do not know mechanisms and routes by which we are accessing the circuitry such as core primitives and rhythm generation through ISMS. We also currently do not know with certainty if this access is truly robust, repeatable across different individuals and easily controlled in behaving mammalian spinal cords. ISMS remains interesting both as a probe to perturb spinal systems in neurobiology, and as a possible neuroprosthetic device basis. However, there are also several known concerns with intraspinal stimulation methods and multiple sets of unknowns.

## What is the Ideal ISMS Device?–How Does ISMS Work in Current Practice?

An ideal ISMS or optrode based spinal neuroprosthesis should be easily surgically implanted and stable when in place, and these are likely solvable technical design issues. However, once such a technology exists (unfortunately it does not yet operate robustly), then other concerns will dominate. It will then be critical for neurosurgeons to know what segments and spinal laminae or coordinates to target, to know how many targets are required, and to know what variety of implants are needed. Similarly, the bioengineer will need to understand how to control and provide means to operate the device effectively. Control and superposition of primitives using ISMS may be a way to target and precisely control the motor output. However, the primary difficulties in fully embracing this perspective currently is the limited scope and the ambiguity of the various data support that have been obtained thus far using ISMS, including our frog work. There are many unknowns and some counter examples to moderate our enthusiasm. These gaps are signposts to needed future work.

### What is the stimulated target of ISMS?

First, how does ISMS recruit modules and motor pools? This may differ between different target species due to evolutionary differences (e.g., frog spinal cord is considerably less sophisticated than rats' spinal cord, and rat spinal cord is much smaller than cat or human spinal cord and lacks some types of corticospinal controls). Data collected on how ISMS works in practice have differed. In the frog (Giszter et al., 1993), it was discovered that ISMS results persisted stably after chronic spinalization (i.e., all descending tracts from outside spinal cord had degenerated and could not contribute), and after chronic deafferentation (i.e., all afferent inputs had degenerated and could not contribute), though it was clear in these latter experiments that afferents helped shaped force-field structure somewhat (Giszter et al., 1993; Loeb et al., 1993). In motor pool stimulation used to recruit fractionated responses it was suggested that the primary targets of electrical stimulation are afferents or propriospinal and local interneurons processes rather than motoneurons (Mushahwar et al., 2004; Gaunt et al., 2006), thereby giving the means for more normal recruitment and other benefits (Mushahwar et al., 2002). Similarly, activation of propriospinal (Yakovenko et al., 2007) or upstream spinal circuitry may underlie some recruitment of primitives and patterning circuitry by ISMS. Our deeper understanding of this issue for design of neuroprostheses is limited in part by our knowledge of spinal circuitry. It is estimated there are hundreds of spinal interneuron types, in terms of molecular and developmental genetics, but the known sets of interneurons identified in adult spinal cords currently are much smaller in number. The range of interneuron roles, locations and interactions possible in spinal cord thus remain murky.

### Mapping and targeting issues in ISMS

ISMS has now been performed in rats, cats and monkeys. However, the mapping extents in terms of topography explored, the durations of mapping and the numbers of repetitions of maps obtained have varied. Some of this is due to the severe technical limitations with current electrode systems, and the short lived nature of some of the physiological preparations. However, repeatability and stability of such ISMS maps remain a concern. Further, different physiological preparations can leave the cord in different states. In rat lumbar cord of chronic spinal rats Tresch and Bizzi obtained largely similar results to those seen in frog, but had more difficulty recruiting motoneurons directly (Tresch and Bizzi, 1999). In rat cervical spinal cord in contrast, Sunshine, Moritz and colleagues showed results of ISMS across spinal cord under 2% isoflurane anesthesia were similar both before and after injury and showed clear topography (Sunshine et al., 2013). However, Moritz and colleagues, working in monkey, in contrast to the rats, reported ISMS results elicited under ketamine anesthesia had little systematic and predictable topography (Moritz et al., 2007). Zimmerman and colleagues working with ISMS in monkey did not comment on topography (Zimmermann et al., 2011). The major limitation on extensive mapping across species is the cost, density and lack of stability and durability of current electrode technologies. The Brain Initiative technologies anticipated might significantly further progress in this area. Clearly topography and its variations and stability are key issues for effective devices.

### Superposition issues in ISMS

**Figure 6:**
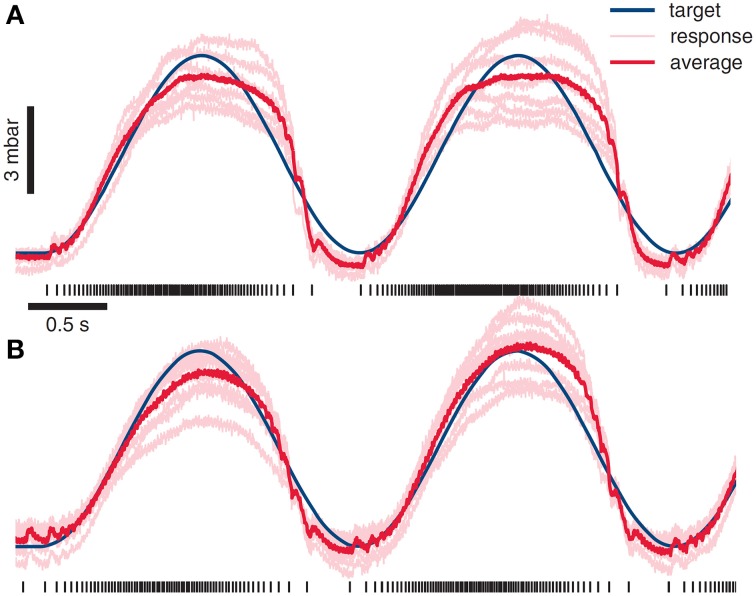
**ISMS stimulus combination used as a basis for driving of force generation and behaviors**. The value of ISMS and modular approaches are the potential simplicity of control and a unified framework for motion and interaction controls. Using a muscle model and ISMS Zimmermann and colleagues have driven grip force using ISMS at sites producing hand grasp motions. *Reproduced from* Zimmermann et al. (2011) with permission. **(A)** An integrate and fire model used in grip force control generates forces with r-squared of 0.92 in relation to desired target force. **(B)** A more sophisticated muscle models allows ISMS control of grip force with r-squared of 0.96.

Zimmerman, Seki and Jackson in monkey, working in a fairly complex anesthetic regime (“induced with ketamine, maintained with isoflourane during surgical dissection, and then switched to an intravenous infusion of propofol/alfentanil to maintain spinal excitability during stimulation experiments”), found that they were able to obtain superposition of EMG and control grip force and limb motion in this way in a primate (Zimmermann et al., 2011). Combination could be used effectively for tracking force or position targets, see Figure 6. Recently they have added BCI control to the ISMS (Zimmermann and Jackson, 2014), as have Nishimura et al. (2013).

In contrast are data in cats. While Lemay and Grill also found a clearly modular synergy organization mapping lumbar spinal cord in unanaesthetized decerebrate cats (Lemay and Grill, 2004), they found co-stimulation effects were exclusively winner-takes-all for all combinations explored. Sequenced activation was tested, and generated motion. However, this motion lacked the range and quality seen in locomotion (Lemay et al., 2009) or in the CPG steplike activations obtained by Barthelemy and colleagues in spinal cats (Barthelemy et al., 2006).

As in the mapping studies results, the ISMS community recognizes these issues, but is limited in approaches and range of tests possible by the current electrode technologies, the stimulation tools and possible electrode or optrode densities.

### Plasticity of ISMS topography issues in ISMS

Lemay and Boyce mapped unanesthetized chronic spinal cats with ISMS, similar to Tresch and Bizzi work in rat. They found that in cats the maps had a topography that differed among individual cats. Maps were shown to be varying based on the cats rehabilitation experience (Boyce and Lemay, 2009). However, the modules they found did not vary among cats, and closely resembled those in Lemay and Grill (2004). Unfortunately, Lemay and Boyce did not test summation and co-stimulation effects in these cat spinal preparations for time reasons. Rhythm generation has also been elicited by ISMS approaches, and is well characterized and mapped in spinal cats in Barthelemy et al. (2006, 2007). Barthelmy and colleagues also saw changes in numbers of sites eliciting pattern generation and stepping effects between the cords of trained and untrained spinal cats. Chronic ISMS in awake spinalized rats and cats has been tested by Mushahwar and colleagues (Bamford et al., 2005, 2010, 2011; Guevremont et al., 2006, 2007; Holinski et al., 2011, 2013) but interpretation has been limited by the electrode issues and the small number of sites and lack of stability of threshold often observed at tested sites in chronic animals. ISMS can also clearly of itself promote spinal plasticity (Kasten et al., 2013) just as do the epidural approaches to spinal stimulation.

Taken together, the data in mammals suggest that the injury type, the rehabilitation experience, the type of anesthesia, and possibly the species and whether the cervical or lumbar enlargement were tested can all contribute to the precise types of ISMS results found. All the authors quoted above were positive about the possibilities of ISMS. However, to move ISMS in a clinical direction with confidence will require much more extensive testing, and assessments of the topography and levels of stability of sites. We need further experience with, and understanding of the interaction of the stimulation effects and combination rules with the type of injury, rehabilitation and other factors in the different animal models (e.g., different therapeutic drugs, viral treatments, and implant types). Clearly, excellent long-lived chronic electrodes that allow rapid multisite switching and recording are again key for exploring these issues.

### Non-linear state dependent effects in ISMS

Our experience is that even in the lowly frog there are bilateral interactions in ISMS co-stimulation that may be non-linear (Giszter et al., 2001). Further, in spinal frogs we have observed, though not fully explored, clear non-linear interactions between ISMS and an ongoing reflex behavior. This interaction is clearly important if intrinsic reflexes or triggered rhythms occur in combination with ISMS layered over them for more precise controls. Most ISMS maps in Section *Mapping and Targeting Issues in ISMS*, and combination tests in Section *Superposition Issues in ISMS* were performed in quiescent or resting spinal cord. It is conceivable cord state changes in behavior alter susceptibility and effect of ISMS at many sites. Although the spinal systems easily combine primitives during behavior, and in corrections, we have been forced to conclude that the bioengineer designing an effective ISMS regime is not guaranteed such an ability. Again, the unanesthetized spinal frog may show significant state dependent response changes to ISMS effects based on activation of reflex behaviors. Thus, far, such issues in exploiting ISMS in mammals are completely unexplored. Most researchers have routinely examined ISMS functional effects while working from a quiescent cord baseline state, and stayed restricted to a hemicord rather than considering bilateral coordination and control issues. This is in part a limitation imposed by the constraints of preparation stability and longevity that are currently possible even with state of the art acute and chronic electrodes. It is also due to a experimenter's natural focus on initially keeping tasks simple for a neuroprosthetic control. However, to an extent, this limitation on published experiments may also have precluded carefully trying to analyze the issues likely to be encountered in fully dynamic experiments and interlimb controls.

### Out of domain side effects in ISMS

Among other ISMS applications that have also been considered in mammals, in addition to limb control, are assisting bladder emptying, continence and other controls (see Tai et al., 1998, 2000, 2001, 2004; Grill and Kirsch, 1999; Grill et al., 1999, 2001; Jezernik et al., 2002; Pikov and McCreery, 2004; Pikov et al., 2007). ISMS may also has a significant role to play in future management of autonomic functions in disease states, and “electroceuticals” or bioelectric therapies (Famm et al., 2013; Birmingham et al., 2014), and it is important to consider that the many issues in segmental motor control using ISMS are likely to also apply to autonomic functions. Further, at the spinal segmental level these two classes of functions may be intimately interwoven and coordinated (e.g., see Zimmerman et al., 2012). The ISMS community currently largely ignores the tight interweaving of autonomic and spinal sensory and motor control systems that the spinal cord may manage. In the intact animal these are integrated seamlessly and without much volitional effort. ISMS autonomic side effects in motor prostheses are largely neglected. Similarly, as optogenetic stimulation(Alilain et al., 2008; Alilain and Silver, 2009) or inhibition with intraspinal techniques are added (Caggiano et al., 2014) autonomic side effects or linked controls may be of concern and remain a major gap in knowledge. The issues in this area are in part that currently short duration experiments on ISMS could have long-term effects in say autonomic control. Understanding these will require more stable and long-term recording, stimulation and monitoring systems. Further, there are similar significant limitations on current autonomic recording techniques to other areas. These are active areas of technology development and potentially of great relevance to the ISMS community.

In summary, as new technologies for recording and stimulation that allow testing of chronic interactions in dynamic contexts are introduced, many of the ISMS issues enumerated here will become manageable. The new neurotechnologies anticipated will hopefully allow areas to be explored, identified and estimated with much greater precision than the community's current sketches of spinal stimulation effects to date have allowed.

## A program of ISMS research based on the current “known unknowns”

Taking into consideration the brief review above, and the gaps in knowledge highlighted by the contrasting results obtained, it seems useful to outline several key areas as targets for future work. Likely within the near future a battery of novel electrode technologies enabling deeper and more extensive stimulation, recording and longer term experiments will become available. What are the pre-clinical priorities to best gather information on and manage device development before human deployment, based on Section *What is the Ideal ISMS Device?–How Does ISMS Work in Current Practice?* The areas of focus below may be in common for each of the available ways of activating spinal cord locally, including ISMS, focal uncaging, iontophoresis of transmitters, and optogenetics approaches. In the future the ISMS and spinal optogenetic neuroprosthetics communities will likely need to populate these focus areas of “known unknowns” with data from a range of comparative animal studies. These same issues may also be areas for focused pre-clinical consideration for translation of intraspinal neuroprosthetic devices. Broadly speaking, and unfortunately, we currently know very little about any of the following issues.

### Topography and threshold ISMS across the segmental motor system, the topography of autonomic recruitment by ISMS, and linkages with motor effects

Spinal cords have been mapped with ISMS to varying extents, often under different forms of anesthesia. A working clinical neuroprosthetic device will operate under no anesthesia, though potentially with local neuromodulation added (e.g., Courtine et al., 2009), and other therapeutic agents on board. Testing and mapping should thus ideally occur under target operating conditions of modulation and no anesthesia. This will be essential for effective translation and comparison across the different models. Repeatability of maps *among individuals* and more especially *over time* should be assessed thoroughly, especially if sufficiently robust and precise chronic implant technology becomes available.

### Compositional rules for ISMS motor responses across the fullest range of target spinal topographies

Combination effects in ISMS have rarely been thoroughly explored. There are many gaps in our knowledge. These run from incompletely explored to completely unexplored issues in combination and interaction of cord stimuli. Particularly relevant to locomotion and bimanual control tasks is including bilateral interactions. Bilateral interactions can be significant. In the rare instances that these have been tested this issue has clearly ruled out some sites as good ISMS targets for simpler neuroprosthetic controls based on strict and simple superposition rules (see Giszter et al., 2001). It is also important to explore and understand the combination interactions of the cervical and lumbar enlargements for ISMS. Are there regions of spinal cord that give robust modular responses and fully predictable superposition with stimulation at all other distant sites, or is combination and superposition elicited by ISMS highly contingent on the presence or absence of stimulation at distant sites? We must move well beyond the simpler two way tests of local combinations within a lumbar or cervical hemicord for effective translation. Ideally, we should also monitor autonomic function in these tests.

### Compositional rules for ISMS depending on cord state and in interactions with naturally elicited behaviors

We need to identify and understand how systems identified in Sections *Topography and Threshold ISMS across the Segmental Motor System, the Topography of Autonomic Recruitment by ISMS, and Linkages with Motor Effects* and section *Compositional Rules for ISMS Motor Responses across the Fullest Range of Target Spinal Topographies* are altered by cord state changes occurring during behavior. This is needed for various reasons. Can ISMS neuroprosthetic controls be effectively and consistently integrated with spared and residual function, e.g., in SCI or other disease states? Specifically, what are ISMS site interactions with pattern generator recruitment; what are the effects on ISMS responses, thresholds and combinations of naturally recruited reflex, rhythmic and voluntary responses acting in parallel to ISMS? Although much of this is unknown, the results with epidural stimulation interactions with cord controlled through external biomechanical states are encouraging (Hsieh and Giszter, 2011; Wenger et al., 2014). However, it also only fair to note that these epidural effects may operate in part through afferent fiber recruitments and access the cord circuitry quite differently from deeper and more focal ISMS. Further, plasticity may eventually impact stimulation patterns used in such chronic successful applications (Angeli et al., 2014; Sayenko et al., 2014).

### Topographies and compositional rules for ISMS motor responses in different spinal cord lesion states, in disease states, and following rehabilitation

The effects and structures identified in intact spinal cords, and the compositional rules and superposition of ISMS effects described as concerns in Sections *Topography and Threshold ISMS across the Segmental Motor System, the Topography of Autonomic Recruitment by ISMS, and Linkages with Motor Effects, Compositional Rules for ISMS Motor Responses across the Fullest Range of Target Spinal Topographies*, and *Compositional Rules for ISMS Depending on Cord State and in Interactions with Naturally Elicited Behaviors* may also potentially be radically different in disease states or after differing degrees of trauma and differing losses of inputs. Most work, with notable exceptions (Kasten et al., 2013) have only explored complete and clean spinal transection at a single level, or intact cords in decerebrate or anesthetized states. While the work of Moritz and colleagues supports a fairly stable set of responses after SCI, the work of Lemay and Boyce suggests the maps can be altered by the rehabilitation compensations and plasticity induced by training. Similarly organic changes in expressed EMG modularity were observed in stroke and SCI by several authors in human clinical settings without any prosthetics (Cheung et al., 2009; Clark et al., 2010; Cheung et al., 2012). These are presumably also reflected in cord changes that likely effect ISMS responses, modular recruitment and combination effects etc. ISMS itself might be expected to progressively alter cord state, and thus change its own effects over time, as has been sometimes observed in clinical epidural stimulation effects.

Modularity is both a potential tool for ISMS but can be a constraint if responses are limited to compositionality drawn exclusively from the spinal modular domain. For example, ISMS recruitment of modular mechanisms in cervical enlargement for the upper limb and hand controls is unlikely to restore precise corticospinally mediated precision grips (Gentner and Classen, 2006; Valero-Cuevas, 2009; Racz et al., 2012). The cord may need to be accessed at the muscle and motor pool level in such tasks, perhaps with the added complexity of actively avoiding or suppressing engagement of grasp reflex circuits that might interfere with such a precise and fine controlled task. Accessing the spinal cord with ISMS at all sites in Figure 5 including motor pools might be important for very fine grained controls. However, it must be acknowledged that demonstrations of BMI control through ISMS without any specific reference to modularity are also clearly feasible (Shanechi et al., 2014). Further, in such contexts, in incomplete lesions, ISMS might also be able to provide more precisely the kinds of functions possible with epidural stimulation (Angeli et al., 2014; Sayenko et al., 2014). ISMS thus might also be feasibly used for supporting and enhancing cortical and voluntary access to spinal cord, presumably in a non-modular fashion. To date, this has not been sufficiently explored in animal models or in as much depth as is warranted.

## Future prospects and anticipating the “known unknowns”

Although a fairly extensive “laundry list” of much needed data is laid out in Sections *Topography and Threshold ISMS across the Segmental Motor System, the Topography of Autonomic Recruitment by ISMS, and Linkages with Motor Effects, Compositional Rules for ISMS Motor Responses across the Fullest Range of Target Spinal Topographies, Compositional Rules for ISMS Depending on Cord State and in Interactions with Naturally Elicited Behaviors, and Topographies and Compositional Rules for ISMS Motor Responses in Different Spinal Cord Lesion States, in Disease States, and Following Rehabilitation*, nonetheless much of the suggested acute work is possible, though not always easy, with current intraspinal stimulation technologies. Studies requiring robust chronic high density ISMS and recording are much more problematic. Chronic and longer term experiments are needed in order to understand the new spinal organization and plasticity issues arising in chronic injury conditions and likely also needed to more fully explore interaction effects, which may require many thousands of combinations and tests to estimate and validate controls and compositional rules, and thus long-lived preparations and experiments. Most of our in depth knowledge of electrode and tissue response comes from electrodes implanted in brain (e.g., Prasad et al., 2014). Data in spinal cord of primate from Nishimura et al. (2013) and in cat from Mushahwar et al. (2000) suggest that current electrodes cannot exceed about 3 month survival times in spinal implantations. The ideal type and design of electrode to record and stimulate spinal cord for long-term neuroprostheses use remains an unsolved problem. Our own effort in this direction has been based around braided electrode technologies (Kim et al., 2013). These were developed initially for answering neurobiological questions, but may support neuroprostheses in the future. As we noted above, the degrees of bending, torsion and motions experienced in the spinal cord make it the most hostile CNS environment for electrodes. We have hypothesized that braided multi-electrode probe (BMEP) designs, which allow open lattice probes with much higher compliance bodies and tethers will be necessary to achieve true longevity in implanted multielectrode neuroprosthetics. Although not easy to deploy currently, these braided designs can support spinal recordings. They exceed compliance of standard microwires significantly (Kim et al., 2013). We expect to improve these further with incorporation of novel materials thereby further reducing the component material modulus (e.g., shape memory polymer approaches see Avendano-Bolivar et al., 2013; Simon et al., 2013; Ware et al., 2013, 2014), We believe that the braid properties of highly compliant and open structures will be especially important in order to cope with the degrees of motion and strain experienced in spinal cord (Kim et al., 2013). These braided probes might allow both recording and stimulation. It may be extremely useful to have both in a neuroprosthetic device in order to manage some of the potential issues noted in Section *What is the Ideal ISMS Device?–How Does ISMS Work in Current Practice?* They might also be combined with other innovations like the FLAME strategy (Abdo et al., 2009; Abdo and Sahin, 2011; Abdo et al., 2011), and a range of other approaches including optogenetics relevant materials (see Caggiano et al., 2014; Lu et al., 2014).

At this point, for neurobiological experiments we need to manage ISMS controls and neural recording data from spinal cord. We are actively anticipating the eventuality that the simpler superposition rules seen in the quiescent frog spinal cord may fail to hold during ongoing behaviors elicited by prior ISMS or reflexes. Rules of superposition of ISMS may also alter or become more variable in the event that the responses are more complex still when tested in injured mammalian spinal cords which are exhibiting behaviors and rehabilitation processes within damaged circuitry.

A probabilistic approach to superposition and spinal cord and limb state may be needed to manage the complexity of the fully behaving or the injured spinal cord. One approach developed by Dr. Sanger of USC that we believe is very promising to manage these issues would abandon the notion of finding simple fully repeatable superposition rules in the ISMS data. It would instead focus on a framework of stochastic control and dynamics (Sanger, 2010, 2011, 2014). Using a combination of biomechanical and neural state from recordings, ISMS patterns that provide an appropriate risk aware stochastic control can be effectively constructed in his framework, using the developed theory of stochastic dynamic operators so as to organize controls in a probabilistic fashion. This framework naturally handles the simple linear superposition results of resting spinal cord as special cases, but can also extend to the more complex control conditions elaborated as potential issues in Section *A Program of ISMS Research Based on the Current “Known Unknowns.”* The probabilistic framework can in principle be updated to manage plastic changing behaviors on longer time scales. Additionally, even when stochastic control is unnecessary for the direct ISMS effects, the stochastic dynamic operator framework can instead provide enhanced predictive data from the controlled spinal cord, to anticipate any parallel behaviors of the spinal circuits acting autonomously through CPG circuitry, elicited following stimulation with altered probability, but that are not directly controlled or driven by the neuroprosthetic control scheme. We therefore believe the Sanger control and analysis framework is likely to be an important addition in ISMS experiments. It may assist in planning the development of effective spinal neuroprosthetics given the landscape of “known unknowns” that we can currently identify ahead. Preparing probabilistic strategies for the worst case scenarios of shifting ISMS topographies and behaviorally varying susceptibility of spinal cord to ISMS and non-linear interactions at a site seems prudent.

## Conclusions

I have tried to present a succinct, but not bias free, summary of the neurobiological data supporting spinal modularity and superposition mechanisms in the spinal construction of movement. This neurobiological perspective largely began with ISMS data, though it has moved well beyond at this point. The framework of pattern generators and primitives as we now understand it both supports and challenges ISMS and optogenetic based intraspinal prostheses. There are reasonably well-defined and modular circuits, but the simpler topographic views of these, as simple target maps in relation to ISMS, may be harder to be confident of currently. A range of new data from a variety of species using ISMS have raised as many questions as they have answered. These have allowed a cursory identification of several important open areas and questions. These issues must be addressed and answered in order to construct an effective intraspinal neuroprosthetic control. Since we do not know how these issues may resolve, we have argued here that it may be prudent to choose control frameworks that manage both guaranteed and probabilistic ISMS results and ISMS superpositions with equal alacrity. At the core of effective intraspinal neuroprosthetics development, as with peripheral interfaces, brain machine interface, and general bioelectronic therapies, the most limiting factors in ISMS advancement remains robust interface design and interface capabilities. In spinal cord these electrode and interface stability issues are particularly severe problems. Many of the known unknowns we have enumerated here will remain so, impeding clinical translation, without a next generation of greatly improved spinal implant designs.

### Conflict of interest statement

We have intellectual property interests in braided electrode designs. The authors declare that the research was conducted in the absence of any commercial or financial relationships that could be construed as a potential conflict of interest.
